# Supplementary material for: SSR marker-based genetic diversity and structure analyses of Camellia nitidissima var. phaeopubisperma from different populations
Source: PeerJ. 2025 Jan 21;13:e18845. doi: 10.7717/peerj.18845 (PMC11758913; doi:10.7717/peerj.18845)
Supplement: Supplemental Information 1 [file peerj-13-18845-s001.docx]

**Appendix 1 DNA extraction procedure**

1. Add 500 μL of lysis solution, an appropriate amount of plant tissue, 100 μL of antioxidant, and 10 μL of RnaseA into a tissue grinding tube, place the tube in the grinder to homogenize the mixture, and incubate at 65℃ for 10 min, during which the tube can be shaken 2 to 3 times;
2. Add 150 μL of polysaccharide scavenger into the grinding tube, mix gently for 15-20 times, and leave at room temperature for 3-5min;
3. Centrifuge at 12000 rpm for 5min at 3.4℃, and transfer 400 μL of supernatant to an extraction plate;
4. Add equal volume of isopropyl alcohol to the extraction plate, and place the extraction plate at Position 1 of the extractor;
5. Add 100 μL of magnetic beads into each well of another extraction plate and place this plate at Position 2, add 500 μL of ethanol into each well of another extraction plate in triplicate and place the three plates at Positions 3, 4, and 5, respectively, and add 100 μL/ 80 μL/ 60 μL (according to the sample status) of eluent into each well of a different extraction plate and place it at Position 6, then run the program after checking instrument status and extracting plate information;
6. After running, take out the extraction plates for DNA testing and store at 4℃.
