# Supplementary material for: SSR marker-based genetic diversity and structure analyses of Camellia nitidissima var. phaeopubisperma from different populations
Source: PeerJ. 2025 Jan 21;13:e18845. doi: 10.7717/peerj.18845 (PMC11758913; doi:10.7717/peerj.18845)
Supplement: Supplemental Information 2 [file peerj-13-18845-s002.docx]

**Appendix 2** **Specifics about sample preparation for capillary electrophoresis**

| Reagent | Volume (μL) |
| --- | --- |
| Fluorescent PCR product | 1.0 |
| GeneScan™500 LIZ | 0.5 |
| Hi-Di™ Formamide | 8.5 |
| Total | 10.0 |

**The system and procedure of M13 linker method**

System:

Mix 5 µl

PrimerF(10 pm) 0.1 µl

PrimerR(10 pm) 0.4 µl

PrimerM13(10 pm) 0.4 µl

ddH2O Add until 9 µl

DNA template 1 µl

Procedure:

Initial denaturation at 96 ℃ for 5 min → (denaturation at 96 ℃ for 30 sec → anneal at 62-52℃ for 30 sec → extension at 72℃ for 30 sec）X 10 cycles→(denaturation at 96 ℃ for 30 sec → anneal at 52℃ for 30 sec → extension at 72℃ for 30 sec)X 20cycles → 72℃20min → 4℃ forever.

Note: For annealing at 62-52°C, start from 62°C and temperature drops by 1℃ in every subsequent cycle until reaching 52°C.
