# Supplementary material for: SSR marker-based genetic diversity and structure analyses of Camellia nitidissima var. phaeopubisperma from different populations
Source: PeerJ. 2025 Jan 21;13:e18845. doi: 10.7717/peerj.18845 (PMC11758913; doi:10.7717/peerj.18845)
Supplement: Supplemental Information 4 [file peerj-13-18845-s004.docx]

**Table S2 Soil composition at 14 sampling sites.**

| **Site** | **PH** | **OrganicMatter (mg/g)** | **Total Nitrogen (mg/kg)** | **Total Phosphorous (mg/kg)** | **Total Potassium (mg/g)** | **AvailableNitrogen (mg/kg)** | **Available Phosphorous (μmol/ml)** | **Available Potassium (mg/kg)** | **Exchange-able calcium (mg/kg)** | **Exchangeable Magnesium (mg/kg)** | **Exchange-able Iron (mg/kg)** | **Exchange -able Copper (mg/kg)** | **Exchangeable Manganese (mg/kg)** | **Exchangeable Zinc (mg/kg)** | **Exchangeable Boron (mg/kg)** |
| --- | --- | --- | --- | --- | --- | --- | --- | --- | --- | --- | --- | --- | --- | --- | --- |
| **CF** | 4.793± 0.214cde | 0.940± 0.081fg | 857.204±40.230ef | 553.854± 7.053fg | 18.031± 0.771g | 90.0135±4.3616de | 0.348±0.017 h | 53.885± 0.883hi | 734.469± 47.904e | 754.463± 34.801a | 8.293± 0.321c | 5.411±0.234gh | 16.316±0.723a | 3.015±0.156 bc | 0.063±0.001b |
| **LP** | 4.3633± 0.193e | 1.257± 0.051c | 1007.720±21.259 bc | 583.528± 7.834de | 23.812± 1.258cd | 109.4542±2.849b | 0.468±0.009ef | 155.323± 5.727cd | 811.642± 49.600cd | 533.818± 22.917g | 8.538± 0.244c | 6.108±0.309bcd | 12.455±1.063de | 2.855±0.081 cd | 0.047±0.002d |
| **CT** | 4.723± 0.127de | 0.487± 0.006h | 449.737±20.466i | 555.395± 11.491fg | 16.678± 1.178g | 65.1489±4.6333f | 0.402±0.016gh | 49.198± 2.085i | 770.170± 33.019de | 666.787± 18.860bcd | 9.651± 0.284b | 5.586± 0.319fg | 16.893± 0.751a | 3.468± 0.109a | 0.044±0.003 def |
| **HP** | 4.997± 0.163bcd | 1.332± 0.069bc | 1165.996±68.684a | 576.083± 4.496ef | 18.342± 0.447g | 121.0153±4.7337a | 0.451±0.013fg | 63.982± 2.917gh | 1019.805±27.747a | 594.230± 16.580f | 8.608± 0.810c | 6.066±0.297bcde | 11.376±0.490fg | 3.106±0.174b | 0.044±0.002 def |
| **WZ** | 5.733± 0.163a | 0.948± 0.054fg | 825.087±45.165f | 569.690± 7.095efg | 28.426± 1.811b | 93.9850±3.4849d | 0.398±0.026gh | 197.366± 12.765b | 889.111± 2.759b | 618.684± 42.979ef | 5.789± 0.355e | 4.834±0.156i | 14.940±0.531b | 2.596±0.115e | 0.056±0.002c |
| **CZ** | 5.017± 0.249bcd | 0.646± 0.021i | 571.647±5.650h | 604.639± 15.854d | 17.124± 1.023g | 70.8536±4.3789f | 0.519±0.025de | 56.594± 4.280hi | 752.727± 32.785de | 640.721± 45.316cde | 10.568± 0.816a | 5.698±0.174efg | 10.143±0.377h | 2.950±0.223 bc | 0.046±0.003 de |
| **SL** | 4.487± 0.125de | 1.042± 0.067e | 869.540±37.358ef | 602.515± 15.049d | 17.471± 1.500g | 97.0782±7.1277cd | 0.538±0.021d | 54.921± 2.518hi | 836.385± 19.151bc | 626.031± 29.635ef | 7.411± 0.269d | 6.673±0.410a | 10.420±0.413 gh | 2.628±0.125d | 0.055±0.002c |
| **WM** | 4.620± 0.125de | 0.915± 0.022g | 841.587±22.308f | 697.417± 29.685c | 21.977± 1.434de | 92.2777±3.6262d | 0.854±0.057c | 110.839± 8.311f | 584.824± 18.187f | 629.483± 12.435def | 8.086± 0.573cd | 5.536±0.141 fg | 14.246±0.119bc | 2.709±0.182d | 0.059±0.003b |
| **SK** | 5.807± 0.253a | 1.132± 0.025d | 971.423±104.106 cd | 788.125± 6.708b | 18.753± 0.681fg | 105.1396±8.6046b | 1.130±0.056b | 71.883± 2.288g | 879.483± 57.120b | 614.010± 23.552ef | 10.219± 0.045ab | 6.392±0.088a | 14.231±0.265bc | 3.012±0.136 bc | 0.046±0.002 de |
| **GG** | 5.033± 0.205bcd | 1.434± 0.018a | 1173.321±86.556a | 600.462± 9.733d | 23.788± 0.886cd | 125.0230±4.7102a | 0.522±0.018de | 152.312± 11.910d | 888.411± 51.582b | 531.482± 21.584g | 5.042± 0.041f | 4.831±0.081i | 11.521±0.701ef | 2.607±0.152e | 0.037±0.002g |
| **BB** | 4.400± 0.243e | 1.011± 0.055ef | 926.480±41.619de | 546.274± 12.668g | 22.552± 1.353de | 94.6682±2.8864d | 0.354±0.026 h | 124.688± 5.626e | 770.683± 28.429de | 694.728± 18.212b | 8.250± 0.582c | 5.029±0.3065hi | 12.7188±0.6754d | 2.8506±0.17802cd | 0.0433±0.00216def |
| **XA** | 5.310± 0.121abc | 1.3573±0.090ab | 1065.820±45.321b | 1117.494± 40.490a | 20.879± 1.213ef | 118.8171±5.8725a | 2.281±0.130a | 101.224± 0.950f | 1024.235±69.720a | 544.535± 21.976g | 9.921± 0.329ab | 6.261±0.046bc | 13.882±0.914c | 2.563±0.105e | 0.041±0.001f |
| **HZ** | 5.543± 1.065ab | 1.161± 0.048d | 1007.216±68.336 bc | 569.695± 2.459efg | 31.402± 1.988a | 103.952± 5.835bc | 0.408±0.013 fgh | 247.859± 16.776a | 784.902± 27.654cde | 670.444± 21.997bc | 10.014± 0.613ab | 5.732±0.451defg | 17.277±0.870a | 3.025±0.137 bc | 0.070±0.004a |
| **RL** | 5.373± 0.352ab | 0.826± 0.037h | 699.223±41.461g | 585.705± 11.501de | 25.717± 2.732c | 83.288± 4.163e | 0.458±0.021 efg | 166.532± 11.436c | 835.192± 33.499bc | 649.678± 8.914cde | 5.767± 0.114e | 5.925± 0.204cdef | 13.719±0.574c | 3.042±0.063 bc | 0.043±0.001ef |

**Note:** Here the site names on the first column are abbreviations of the sampling sites, please see Table 1 for the specific locations.
