# Supplementary material for: SSR marker-based genetic diversity and structure analyses of Camellia nitidissima var. phaeopubisperma from different populations
Source: PeerJ. 2025 Jan 21;13:e18845. doi: 10.7717/peerj.18845 (PMC11758913; doi:10.7717/peerj.18845)
Supplement: Supplemental Information 6 [file peerj-13-18845-s006.docx]

**Table S4 PCR amplification procedure for** ***Camellia nitidissima* var. *phaeopubisperma***

| **Reagent** | **Volume (μL)** | **Process** | **Time** | **Cycles** |
| --- | --- | --- | --- | --- |
| 2×Taq PCR Master Mix | 5.0 | Initial denaturation at 95℃ | 5min |  |
|  |  | Denaturation at 95℃ | 30sec | 10 cycles; temperature drops by 1℃ in every subsequent cycle |
| Genome DNA (~20ng) | 1.0 | Anneal at 62℃-52℃ | 30sec |  |
|  |  | Extension at 72℃ | 30sec |  |
| Forward Primer (10pmol/μL) | 0.5 | Denaturation at 95℃ | 30sec | 25 cycles |
| Reverse Primer (10pmol/μL) | 0.5 | Anneal at 52℃ | 30sec |  |
| ddH2O | 3.0 | Extension at 72℃ | 30sec |  |
| Total | 10.0 | Final extension at 72℃ | 20min |  |
|  |  | 4℃ |  |  |
